# PBK/TOPK Inhibitor Suppresses the Progression of Prolactinomas

**DOI:** 10.3389/fendo.2021.706909

**Published:** 2022-01-21

**Authors:** Kejing Zhu, Xueting Cheng, Shuman Wang, Hong Zhang, Yu Zhang, Xiong Wang, Yonggang Chen, Jinhu Wu

**Affiliations:** ^1^ Department of Pharmacy, Tongren Hospital Affiliated to Wuhan University, The Third Hospital of Wuhan, Wuhan, China; ^2^ Department of Pharmacy, Xiangyang Central Hospital, Affiliated Hospital of Hubei University of Arts and Science, Xiangyang, China; ^3^ School of Medicine, Xiangyang Polytechnic, Xiangyang, China; ^4^ School of Pharmacy, Hubei University of Chinese Medicine, Wuhan, China; ^5^ The Second Clinical College, Wuhan University, Wuhan, China

**Keywords:** prolactinoma, PRL, TOPK/PBK, p38 MAPK, HI-TOPK-032

## Abstract

**Background:**

Prolactinoma is the most common type of pituitary tumors, and its resultant tumor occupying and hormone disturbance greatly damage the health of patients. In this study, we investigated a protein kinase-PDZ Binding Kinase (PBK)/T-LAK Cell-Originated Protein Kinase (TOPK) as a candidate protein regulating prolactin (PRL) secretion and tumor growth of prolactinomas.

**Methods:**

Downloaded prolactinoma transcriptome dataset from Gene Expression Omnibus (GEO) database, and screened differentially expressed genes (DEGs) between normal pituitary tissues and prolactinoma tissues. Then, Gene ontology (GO) and Kyoto Encyclopedia of Genes and Genomes (KEGG) enrichment analyses of DEGs were performed, a protein-protein interaction (PPI) network was constructed and the hub genes were identified. After a literature search, TOPK was presumed as an candidate target regulating the prolactinoma. We found a specific inhibitor of TOPK to investigate its effects on the proliferation, migration, apoptosis and PRL secretion of pituitary tumor cells. Finally, the regulation of TOPK inhibitor on its downstream target-p38 Mitogen Activated Protein Kinase (p38 MAPK) was detected to explore the potential mechanism.

**Results:**

A total of 361 DEGs were identified, and 20 hub genes were screened out. TOPK inhibitor HI-TOPK-032 could suppress the proliferation & migration and induce apoptosis of pituitary tumor cells *in vitro*, and reduce PRL secretion and tumor growth *in vivo*. HI-TOPK-032 also inhibited the phosphorylation level of the downstream target p38 MAPK, suggesting that TOPK inhibitors regulate the development of prolactinoma by mediating p38 MAPK.

**Conclusion:**

Our study of identification and functional validation of TOPK suggests that this candidate can be a promising molecular target for prolactinoma treatment.

## Introduction

Pituitary adenoma (PA) is one of the most common intracranial tumors, and its morbidity is about 16% as found by autopsy or Nuclear Magnetic Resonance Imaging (1). Prolactinoma is the most frequent type of pituitary tumor and accounts for 40%-60% in all kinds of pituitary tumors (2–4). The typical clinical manifestations of prolactinomas include amenorrhea, galactorrhea, hyperprolactinemia, osteoporosis and infertility in women, as well as mammoplasia, defective ejaculation and impotence in men (5). Invasive prolactinoma is manifested as massive infiltration to the surrounding anatomic structures, which often grows rapidly, and has a big size but poor prognosis. Invasive prolactinoma easily recurs after surgical resection, and is difficultly cured, and its residual tumor even may be aggravated, which greatly threatens the human health (6–9). At present, some progress has been made in the research on the pathogenesis of pituitary tumor, but the fundamental pathogenesis still has not been clarified. Prolactinoma is treated mainly by surgery and drug therapy to improve tumor occupying and hormone disturbance (10). Dopamine agonists are the major first-line therapeutic drugs, including bromocriptine and cabergoline. However, there are such disadvantages as insufficient drug types and single treatment plans (11). Meanwhile, 10%-30% of patients easily become resistant to dopamine agonists (12). For patients resistant to dopamine agonists and unsuitable for surgery, the treatment is still a clinical challenge. Therefore, it is urgently needed to identify the key genes and pathways which play an important role in the occurrence and development of prolactinoma, to help accurate diagnosis and prognosis, and to develop new therapeutic targets.

TOPK, also known as PBK, encodes a serine/threonine protein kinase related to the dual specific mitogen-activated protein kinase kinase (MAPKK) family (13). Several studies in recent years have shown that TOPK is overexpressed in many tumor tissues, and its expression is significantly correlated with the malignancy and poor prognosis of these tumors (14–17). It has been found that TOPK can facilitate the occurrence and development of tumors (e.g., ovarian cancer, malignant glioma, colonic cancer and non-small-cell lung carcinoma) by promoting their growth, invasion and metastasis (18–21), thus it is regarded as a new target for tumor-specific therapy. In fact, TOPK has attracted public concerns as a tumor-specific biomarker and a biochemical target, and it has the tumor-treating potential but a minimal damage to normal tissues (22). TOPK, similar to MKK3/6, is identified as a new member of MAPKK family and has a function of promoting the phosphorylation of p38 MAPK, thus p38 MAPK has been identified as a PBK/TOPK-specific substrate (23). There is a lot of evidence showing that PBK/TOPK could promote the proliferation and migration of tumor cells by activating p38 MAPK (23–25). Our preliminary study has confirmed that p38 MAPK is closely associated with the occurrence and development of prolactinoma (26). Therefore, TOPK may be the upstream action molecule of p38 MAPK to promote the occurrence and development of prolactinoma, and inhibition of TOPK might have anti-prolactinoma effect.

At present, several TOPK-targeted specific inhibitors have been developed: HI-TOPK-032, OTS514 and OTS964. Studies have shown that HI-TOPK-032 can directly inhibit TOPK activity *in vivo* & *in vitro* and suppress the growth and induce the apoptosis of colonic cancer cells, and also can inhibit the neoplasm growth of colonic cancer in the xenotransplantation mouse model (18). Another study demonstrated that targeting PBK/TOPK with HI-TOPK-032 decreased growth and survival of glioma initiating cells *in vitro* and attenuated tumor growth *in vivo* (27). OTS514 and OTS964 have been evaluated in several solid tumors (e.g., ovarian cancer, myeloma and cervical cancer), and play an anti-tumor role by inducing cell cycle arrest and apoptosis (28–30). Some encouraging achievements have been obtained for low-molecular-weight inhibitors of TOPK as independent therapeutic agents *in vivo* and *in vitro*, and the clinical trials of these inhibitors are promising to be conducted in the near future. By inhibiting TOPK *in vivo* and *in vitro*, we explored the effect of HI-TOPK-032 on the proliferation, apoptosis, cell cycle distribution and migration of pituitary tumor cells, and investigated it’s effect on PRL secretion and tumor growth in prolactinoma model rats.

## Materials and Methods

### Microarray Data

Human expression profile dataset GSE119063 which contained 5 prolactioma tissues and 4 normal pituitary tissues was downloaded from the GEO Database (www.ncbi.nlm.nih.gov/geo). The platform used for this dataset is GPL2895 (GE Healthcare/Amersham Biosciences CodeLink Human Whole Genome Bioarray).

### Identification of DEGs

Firstly, the data were standardized with the function “normalize Between Array” in R software. Secondly, the differential expression analysis in the R computing environment using limma package was performed (31), and then DEGs between normal pituitary tissues and prolactinoma tissues were screened with a standard of adj. *p*-value < 0.01 and |log_2_-fold change | ≥ 2.

### GO and KEGG Pathway Analysis

GO (http://www.geneontology.org/) is a common database to annotate genes and their products and recognize the characteristic organisms (32). KEGG (http://www.genome.jp/kegg/Pathway.html) is a bioinformatic database containing biochemical pathways (33). DAVID (http://david.abcc.Ncifcrf.gov/) online software provides a comprehensive set of functional annotation tools for investigators to understand biological significance behind large list of genes. The GO and KEGG enrichment analysis of up/down-regulated DEGs were performed with DAVID database (34). *P* < 0.05 indicated a statistically significant difference, the results were visualized using R software.

### Construction of PPI Network and Selection of Hub Genes

To further explore the mechanism of DEGs regulating the occurrence and development of prolactinoma and establish an interaction of DEGs, a PPI network was predicted by imputing DEGs into STRING online database (http://string-db.org) (35). DEGs with a relation score > 0.04 were considered to be significant and then visualized using Cytoscape software (36). Thereafter, the clustering analysis of PPI network was performed with Molecular Complex Detection (MCODE) Application to screen the key modules (parameter setting: degree cutoff ≥ 2, node score cutoff ≥ 2, K-core ≥ 2, and max depth = 100), and the key nodes (hub genes) in the network were identified using CytoHubba plug-in and on the basis of MCC algorithm. Finally, the enrichment analysis of these hub genes was conducted at Metascape website.

### Cell Culture

Pituitary tumor cell lines MMQ and GH3 were provided by Chinese Academy of Sciences. GH3 was cultured in DMEM medium (Gibco, 12430047) supplemented with 10% fetal bovine serum (FBS; Gibco, 10270-106), and MMQ was cultured in RPMI-1640 medium (Gibco, 31800105) supplemented with 10% FBS (BI, 1928702), in an incubator with 5% CO_2_ at 37°C. MMQ cells secrete prolactin and GH3 cells secrete both growth hormone and prolactin, and they are appropriate cell models used in the study of prolactima (37).

### Western Blot Assay

The protein expressions of TOPK (16110-1-AP, Proteintech), p38 MAPK (AF6456, Affinity), PRL (DF6506, Affinity), Bax (50599-2-Ig, Proteintech) and Bcl-2 (12789-1-AP, Proteintech) in pituitary tumor cells and pituitary tissues were determined by Western Blot assay. The tissue samples or cell samples were treated with RIPA lysate to extract total protein, and protein concentration was determined by BCA method. The proteins were separated by SDS-PAGE and transferred to polyvinylidenedifluoride membranes. The membranes were blocked with 5% skim milk at room temperature for 1 h, then incubated overnight in the primary antibody at 4°C, and incubated in the secondary antibody for 1 h. After TBST elution, electrochemiluminescence (ECL) was used for exposure, and the gray values of protein bands were analyzed by Image J software.

### Quantitative Real-Time Polymerase Chain Reaction (qRT-PCR)

Referring to the manufacturer’s protocol, total RNA was extracted from MMQ cells using E.Z.N.A.^®^Total RNA Kit II (OMEGA, R6934-01). Then, the first-strand cDNA was synthesized with 1 μg total RNA using RevertAid™ First Strand cDNA Synthesis Kit (Thermo, K1622). Thereafter, fluorescence quantitative polymerase chain reaction (qRT-PCR) was performed with SYBR Premix Ex Taq Kit (TaKaRa, RR420A). GAPDH was used as an internal reference, and the relative expression level of target mRNA was calculated using “2^-ΔΔCT^” method. The amplification procedure was below: pre-denaturation at 95°C for 30 sec, denaturation at 95°C for 15 sec, annealing/extension at 60°C for 30 sec, 35 cycles in total. Primers for qRT-PCR were as follows: PRL-F: GGTTTGGTCACAACTCCCATCCC; PRL-R: TGGACAATTTGGCACCTCAGGAAC; GAPDH-F: AGGTCGGAGTCAACGGATTT; GAPDH-R: ATGAAGGGGTCATTGATGGCA.

### Enzyme-Linked Immunosorbent Assay (ELISA)

The PRL concentrations in the MMQ cellular supernatant and rat serum were measured using ELISA Kit (Elabscience, E-EL-R3006). According to the manufacturer’s instruction, 100 μL of standard working solutions or samples were added into 96-well plate and incubated at 37°C for 90 min, then 100 μL of biotinylated antibody were added and incubated for 60 min, and then washed for 3 times. 100 μL of HRP conjugate working solutions were added into each well, then incubated for 30 min, and washed for 5 times. 90 μL of substrate reagents were added into each well and incubated for 30 min, and then 50 μL stop solutions were added to stop the reaction. Finally, the absorbance at 450nm was measured, and the PRL concentration (pg/ml) was calculated.

### PBK/TOPK Inhibitor

TOPK inhibitor HI-TOPK-032 (Lot number: BCP35692) was purchased from BioChemPartner, Shanghai, China. HI-TOPK-032 can specifically inhibit the activity of TOPK kinase *in vitro* and *in vivo*.

### Cell Counting Kit-8 Assay

Cells in logarithmic phase were digested with 0.25% trypsin and inoculated into a 96-well plate at a density of 5000 cells per well, which were cultured at 5% CO_2_ and 37°C for 12 h. Then the cells were treated with 0.1% dimethyl sulfoxide (DMSO) and different concentrations of HI-TOPK-032 (2 μmol/L, 5 μmol/L, 10 μmol/L). After continuous culture for 24 h, 48 h, 72 h, 96 h and 120 h, 10 µL CCK-8 solution was added into each well, which were re-put into the incubator for 3 h, and thereafter the absorbance (optical density, OD) at 450 nm was measured using a microplate reader.

### Colony Formation Assay

The logarithmic phase cells were digested with 0.25% trypsin and blunted into individual cells, which were inoculated into a 6-well microplate by 800 cells per well. After 12 h, cells were treated with 0.1% DMSO and different concentrations of HI-TOPK-032 (2 μmol/L, 5 μmol/L, 10 μmol/L), and then cultured at 37°Cand 5% CO_2_ for 2-3 weeks. When the cell cluster in control group reached 50 cells, the cells were fixed with pure methanol and stained with Giemsa violet. Finally, the number of colonies with 50 cells or more was counted under a microscope.

### Wound Healing Assay

The cells were inoculated into a 6-well plate by 5×10^5^ cells per well. When the cells were fused to 90% density, a 200 μL of tip was used to scratch along the ruler. The cells were washed for three times with PBS, followed by the addition of serum-free medium added, and then treated with 0.1% DMSO and different concentrations of HI-TOPK-032 (2 μmol/L, 5 μmol/L, 10 μmol/L) respectively, and continuously cultured at 37°C and 5% CO_2_. At 0 h and 24 h, the cells were photographed under microscope (HP×100).

### Transwell Assay

The migration assay of pituitary tumor cells was carried out in an uncoated Transwell chamber without serum medium. After 24 h, the cells were fixed with methanol and stained with Giemsa staining solution for 30min. The non-migrated cells at the upper chamber were carefully wiped off with cotton swabs, while migrated cells were photographed and counted in 5 random fields for each well under microscope (HP×100).

### Flow Cytometry for Cell Apoptosis and Cell Cycle

Cells in logarithmic phase were digested with 0.25% trypsin and then inoculated into a 6-well plate. After the cells adhered to the plate, they were treated with 0.1% DMSO and different concentrations of HI-TOPK-032 (2 μmol/L, 5 μmol/L, 10 μmol/L) at 37°Cand 5% CO_2_ for 48 h. Cells were collected, centrifuged and washed with cold PBS, which were followed by staining with AnnexinV-FITC and Propidium Iodide (PI). And then, percentage and number of cells at different states (mainly including early apoptosis, late apoptosis and death) were detected by flow cytometry. In addition, cells were fixed by pre-cooled 75% alcohol, then RNA was removed and PI staining solution was added to detect cell cyclical distribution by flow cytometry.

### Animal Experiment

SPF SD rats (age: 60d; sex: female; body weight: 200-250 g) were provided by the Experimental Animal Center of China Three Gorges University. This study was approved by the Ethics Committee of Tongren Hospital affiliated to Wuhan University. Rats were randomly divided into 3 groups: (A) control group (n = 10), (B) prolactinoma model group (n = 10), (C) HI-TOPK-032 treatment group (n = 10). The estrogen-induced SD rat model of prolactinoma has been extensively used as an animal model of human prolactinoma in a large number of studies (38, 39). Firstly, each rat in group B and C were intraperitoneally injected with 2 ml estradiol benzoate once in every 5 days for total 8 weeks to establish prolactinoma animal models, while group A were intraperitoneally injected with vectors. After successful modeling, the rats in group C were administered with HI-TOPK-032 (10mg/kg) three times per week for a total of 28 days, and vehicles were administered for group A and B. The weight of rats in each group was measured and recorded weekly. At the end of drug administration, all rats were sacrificed with CO_2_ method, and their pituitary tissues were collected for Western Blot assay, and serum samples were collected for ELISA assay.

### Statistical Analysis

Unless specified, all datas were statistically analyzed with SPSS 24 software and presented as mean ± standard deviation (SD). The statistical analysis was performed by one-way analysis of variance. *P* < 0.05 was considered statistically significant, and the plotting was conducted using GraphPad Prism.

## Results

### Identification of DEGs in Prolactinoma Tissues

**Figure 1:**
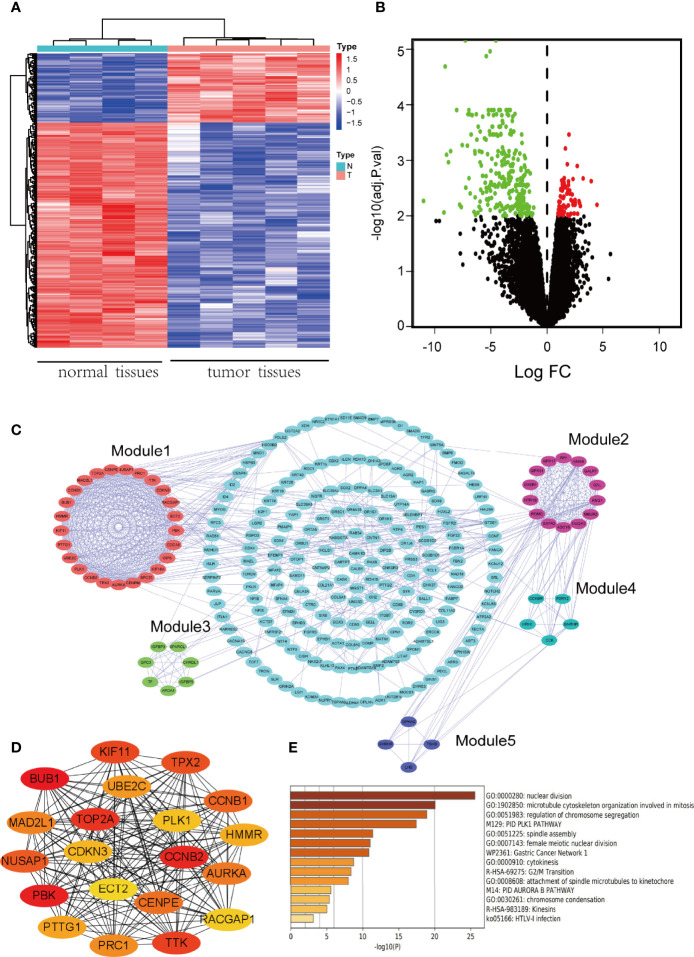
Identification of DEGs and screening of hub genes. **(A)** The heatmap of DEGs. Red represents up-regulated genes, and blue represents down-regulated genes. A total of 361 DEGs were identified. **(B)** Volcano plot of DEGs. Red denotes up-regulated genes and green denotes down-regulated genes (adj. *p*-value < 0.01 and |log_2_FC| ≥ 2). **(C)** PPI network of DEGs. Five modules were identified by MCODE, which were represented by five different colors. **(D)** The network of hub genes which was screened by CytoHubba. The higher the ranking, the darker the color. **(E)** Biofunction and pathway enrichment analysis of hub genes based on Metascape website.

After the standardization of gene chip data, total 361 DEGs were identified by comparing with normal pituitary tissues, including 276 down-regulated DEGs and 85 up-regulated DEGs (|log_2_FC| > 2 and adj. *p*-value < 0.01). The heatmap and volcano plot for the visualization of DEGs were seen in **Figures 1A, B**.

### GO and KEGG Enrichment Analysis

To analyze the biological function of DEGs and better understand its involved signaling pathways, the function and pathway enrichment analysis of DEGs were performed with DAVID database, and the results of GO and KEGG enrichment analysis were visualized using R software. The top 10 GO terms of up-regulated genes and down-regulated genes were shown in **Supplementary Figures 1A, B**. Up-regulated genes were mainly enriched in biological processes, such as chromosome segregation, nuclear division and mitotic nuclear division, while down-regulated genes mainly participate in sensory system development and transmembrane receptor protein serine/threonine kinase signaling pathway. The results of KEGG enrichment analysis were shown in **Supplementary Figures 1C, D**. The KEGG pathway analysis showed that up-regulated genes were significantly enriched in cell cycle and p53 signaling pathway, while down-regulated genes were significantly involved in signaling pathways regulating pluripotency of stem cells and TGF-beta signaling pathway.

### Construction of PPI Network and Screening of Hub Genes

A PPI network of DEGs was constructed by importing the interaction relation data file obtained from STRING database into Cytoscape software. The clustering analysis of PPI network was performed with MCODE plug-in, and 5 modules were selected (**Figure 1C**). The key nodes (hub genes) in the PPI network were identified using CytoHubba plug-in and displayed by networking (**Figure 1D**). According to the MCC algorithm, the top 20 genes were: *BUB1, PBK, CCNB2, TOP2A, TTK, KIF11, TPX2, NUSAP1, CCNB1, CENPE, AURKA, MAD2L1, PRC1, UBE2C, PTTG1, HMMR, CDKN3, PLK1, RACGAP1, ECT2*. These 20 genes were regarded as hub genes because of the highest connection in the network. Finally, the enrichment analysis was conducted for the above mentioned 20 hub genes at Metascape website to further explore their functions (**Figure 1E**). The results showed that hub genes were significantly enriched in GO biological processes, such as nuclear division, microtubule cytoskeleton organization involved in mitosis, regulation of chromosome segregation, and in KEGG pathways, such as G2/M Transition.

### HI-TOPK-032 Inhibited PRL Production in Pituitary Tumor Cells

**Figure 2:**
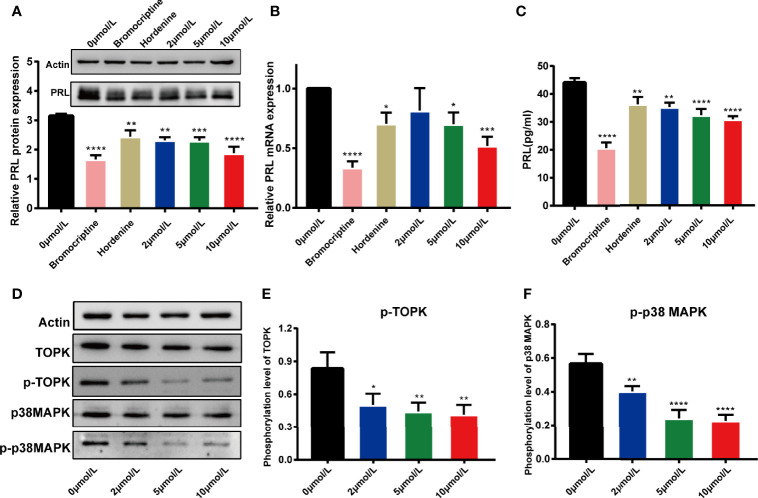
HI-TOPK-032 reduced PRL production in pituitary tumor cells and it worked by mediating p38 MAPK signaling pathway. **(A)** HI-TOPK-032 decreased PRL protein expression in pituitary tumor cells detected by Western Blot assay. **(B)** HI-TOPK-032 decreased PRL mRNA expression in pituitary tumor cells detected by qRT-PCR assay. **(C)** HI-TOPK-032 decreased PRL secretion in cell supernatant detected by Elisa assay. **(D)** The expressions of TOPK, p38 MAPK and its phosphorylation level in pituitary tumor cells after HI-TOPK-032 intervention were detected by Western Blot assay. **(E)** HI-TOPK-032 reduced the phosphorylation of TOPK. **(F)** HI-TOPK-032 reduced the phosphorylation of p38 MAPK. *P < 0.05; **P < 0.01; ***P < 0.001; ****P < 0.0001.

Referring to the potency of bromocriptine (a positive drug) and hordenine (a Chinese herbal monomer preliminarily discovered by our research group), which were both confirmed to have the inhibition on PRL secretion (40), we explored the effect of HI-TOPK-032 on PRL production in MMQ cells. MMQ cells were treated with 0.1% DMSO, bromocriptine (100 μmol/L), hordenine (0.2 mg/ml), as well as 2 μmol/L, 5 μmol/L and 10 μmol/L of HI-TOPK-032. After 48 h, the mRNA & protein expression and secretion levels of PRL were detected by Western Blot assay, qRT-PCR and ELISA. The results suggested that bromocriptine, hordenine, as well as 2 μmol/L, 5 μmol/L and 10 μmol/L HI-TOPK-032 significantly decreased mRNA & protein expression and secretion levels of PRL (*P* < 0.05), as shown in **Figures 2A–C**. HI-TOPK-032 decreased PRL production in a dose-dependent form.

### HI-TOPK-032 Inhibited the Phosphorylation Level of TOPK and p38 MAPK

Recent research findings showed that TOPK could activate its downstream target p38 MAPK. Therefore, inhibiting TOPK activity with HI-TOPK-032 should be capable of reducing phosphorylation of p38 MAPK. We thereby detected the expression of TOPK, p38 MAPK and their phosphorylation levels in HI-TOPK-032-treated MMQ cells. Our results suggested that HI-TOPK-032 can remarkably decrease the phosphorylation levels of TOPK and p38 MAPK (**Figures 2D–F**). Conclusively, HI-TOPK-032 was an effective antagonist of TOPK and can regulate p38 MAPK.

### HI-TOPK-032 Inhibited the Proliferation of Pituitary Tumor Cells

**Figure 3:**
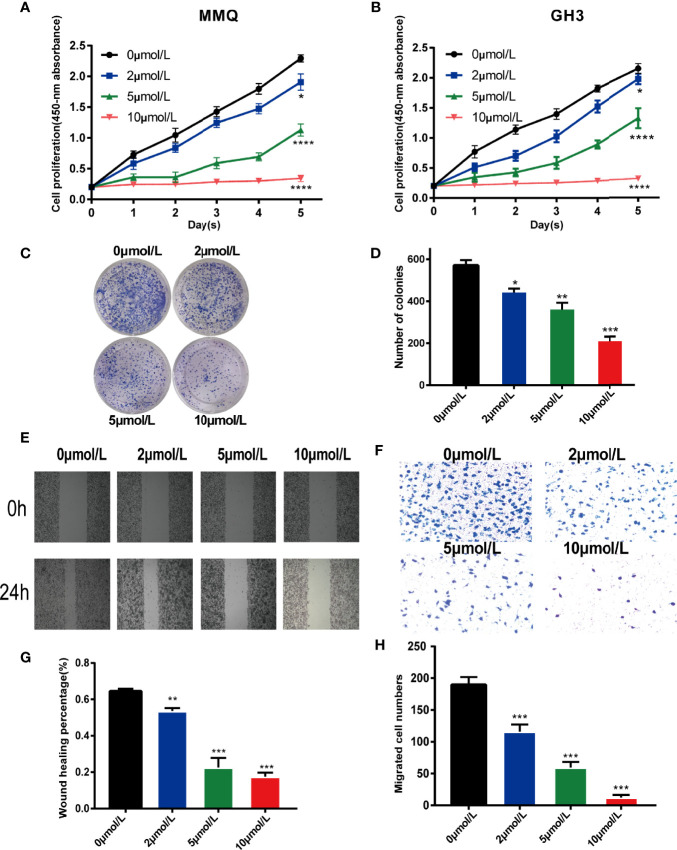
HI-TOPK-032 inhibited the proliferation and migration of pituitary tumor cells. **(A)** The proliferation capability of MMQ cells detected by CCK-8 assay. **(B)** The proliferation capability of GH3 cells detected by CCK-8 assay. **(C)** Cell cloning formation ability measured by plate-based Colony formation assay. **(D)** The number of cell clones was statistically analyzed and histogram was drawn. **(E)** Wound healing assay was used to detect the migration ability of GH3 cells. **(F)** Transwell assay detected the migration ability of GH3 cells. **(G)** Statistical analysis results of Wound healing assay. **(H)** Statistical analysis results of Transwell assay. *P < 0.05; **P < 0.01; ***P < 0.001; ****P < 0.0001.

To explore the effect of TOPK inhibitor on the proliferation capability of pituitary tumor cells, MMQ cells and GH3 cells were treated using 0.1% DMSO (control group, 0 μmol/L) and different concentrations of HI-TOPK-032 (2 μmol/L, 5 μmol/L, 10 μmol/L), and then, the cell proliferation capability was detected by CCK8 assay. At 24 h, 48 h, 72 h, 96 h and 120 h, the OD values at 450 nm of cells in various groups were measured, and the growth curve was plotted (**Figures 3A, B**). The proliferation levels of both MMQ cells and GH3 cells were significantly lower in the treatment group than in the control group (*P* < 0.05), which indicated that TOPK inhibitor could evidently suppress the proliferation capabilities of MMQ cells and GH3 cells. We further validated the effect of TOPK inhibitor on cell cloning ability by Colony formation assay. Compared with the control group, there was a significant decrease in the number of clones when GH3 cells were treated with HI-TOPK-032 (*P* < 0.05; **Figures 3C, D**). It was thereby found that TOPK inhibitor could remarkably inhibit the proliferation and colony formation capabilities of pituitary tumor cells.

### HI-TOPK-032 Inhibited the Migration of Pituitary Tumor Cells

To determine whether HI-TOPK-032 could suppress the migration of pituitary tumor cells, we performed Wound healing assay and Transwell assay. The results of Wound healing assay (**Figures 3E, G**) confirmed that HI-TOPK-032 could inhibit the wound healing speed of pituitary tumor cells (*P* < 0.05). As shown by the results of Transwell assay (**Figures 3F, H**), HI-TOPK-032 could strongly suppress the migration capability of pituitary tumor cells (*P* < 0.05).

### HI-TOPK-032 Induced the Cycle Arrest and Apoptosis of Pituitary Tumor Cells

**Figure 4:**
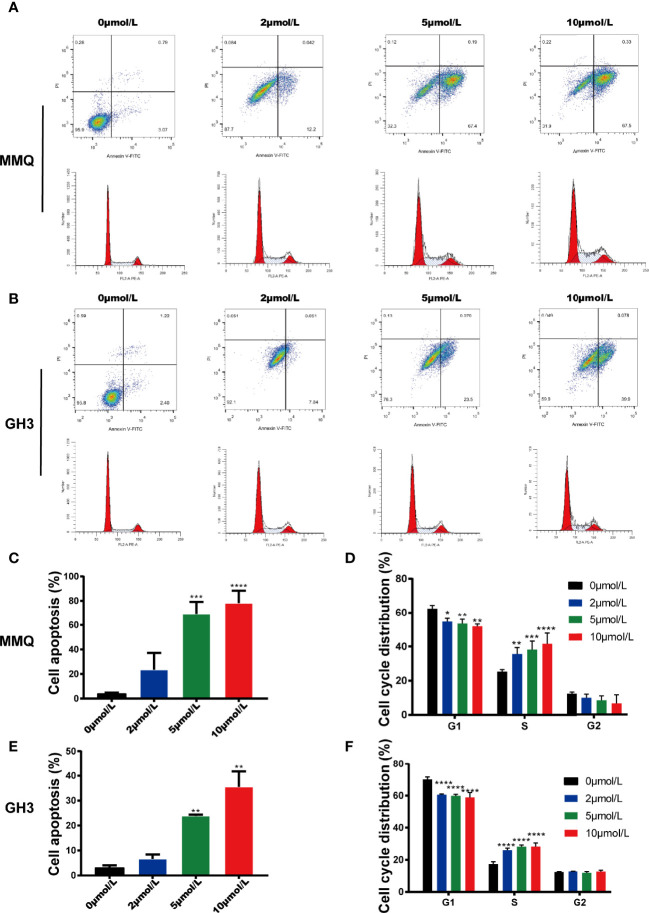
HI-TOPK-032 induced apoptosis and cycle arrest of pituitary tumor cells. **(A)** Flow cytometry detected the apoptosis and cell cycle distribution of MMQ cells after HI-TOPK-032 intervention. **(B)** Flow cytometry detected the apoptosis and cell cycle distribution of GH3 cells after HI-TOPK-032 intervention. **(C)** HI-TOPK-032 induced apoptosis of MMQ cells. **(D)** HI-TOPK-032 down-regulated the percentage of G1 phase and up-regulate the percentage of S phase in MMQ cells. **(E)** HI-TOPK-032 induced apoptosis of GH3 cells. **(F)** HI-TOPK-032 down-regulated the percentage of G1 phase and up-regulate the percentage of S phase in GH3 cells. *P < 0.05; **P < 0.01; ***P < 0.001; ****P < 0.0001.

We further explored the effects of HI-TOPK-032 on the apoptosis and cell cycle distribution of pituitary tumor cells by flow cytometry. At 48 h after MMQ cells and GH3 cells were treated with 2, 5 and 10 μmol/L of HI-TOPK-032, the cell apoptosis and cell cycle distribution were detected. The results showed that 5 μmol/L and 10 μmol/L of HI-TOPK-032 could induce the apoptosis of both MMQ cells and GH3 cells (*P* < 0.05), and HI-TOPK-032 (2 μmol/L, 5 μmol/L, 10 μmol/L) could down-regulate the percentage of G1 phase and up-regulate the percentage of S phase in MMQ and GH3 cells, indicating S phase arrest (**Figure 4**). In addition, we also detected the expression of apoptosis-related proteins Bax and Bcl-2 by Western Blot assay (**Supplementary Figure 2**), and found that 5 μmol/L and 10 μmol/L of HI-TOPK-032 significantly up-regulated Bax expression but down-regulated Bcl-2 expression in MMQ cells and GH3 cells, which further validated the induction of HI-TOPK-032 on the apoptosis of pituitary tumor cells. Therefore, HI-TOPK-032 could induce the apoptosis and S-phase arrest of pituitary tumor cells.

### HI-TOPK-032 Inhibited Prolactinoma Growth and Reduced PRL Secretion *In Vivo*

**Figure 5:**
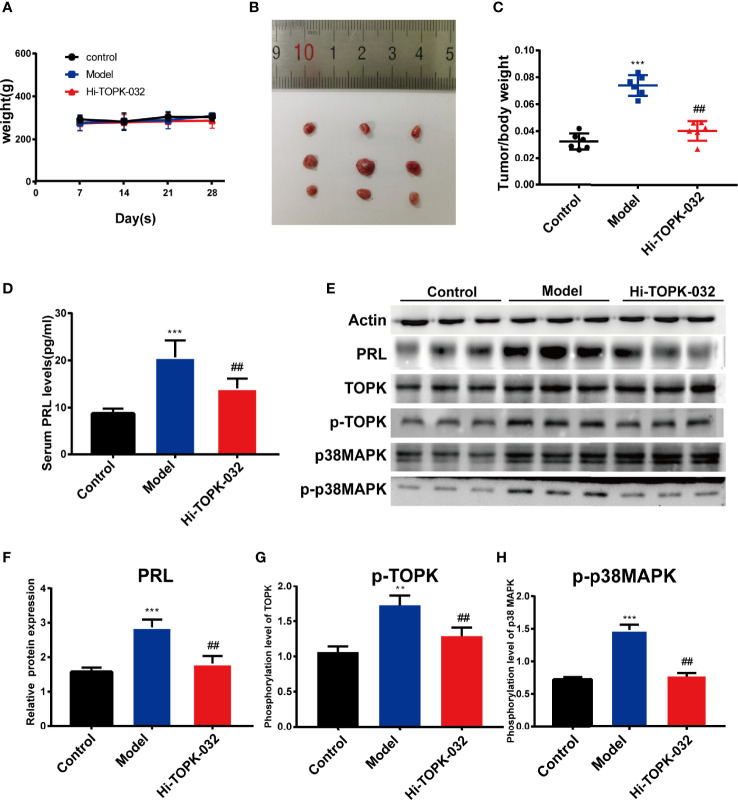
HI-TOPK-032 inhibited tumor growth of prolactinoma and reduced PRL secretion *in vivo*. **(A)** The body weight changes of rats in each group were recorded once per week during treatment. **(B)** Photograph of the pituitary tissues. The first row represents the control group, the second row represents the model group, and the third row represents the HI-TOPK-032 (10mg/kg) treatment group. **(C)** The ratio of pituitary weight to body weight of control group, model group and Hi-TOPK-032 treatment group. **(D)** PRL secretion levels in serum of rats in each group were detected by ELISA assay. **(E)** The protein expressions of PRL, total TOPK, p-TOPK, total p38 MAPK and p-p38 MAPK in pituitary tissues of rats in each group were detected by Western Blot assay. **(F)** The protein expression of PRL. **(G)** Phosphorylation level of TOPK. **(H)** Phosphorylation level of p38 MAPK. **P < 0.01; ***P < 0.001; ^##^P < 0.01; ** and *** represent comparison with the control group; ^##^ means compared to the model group.

**Figure 6:**
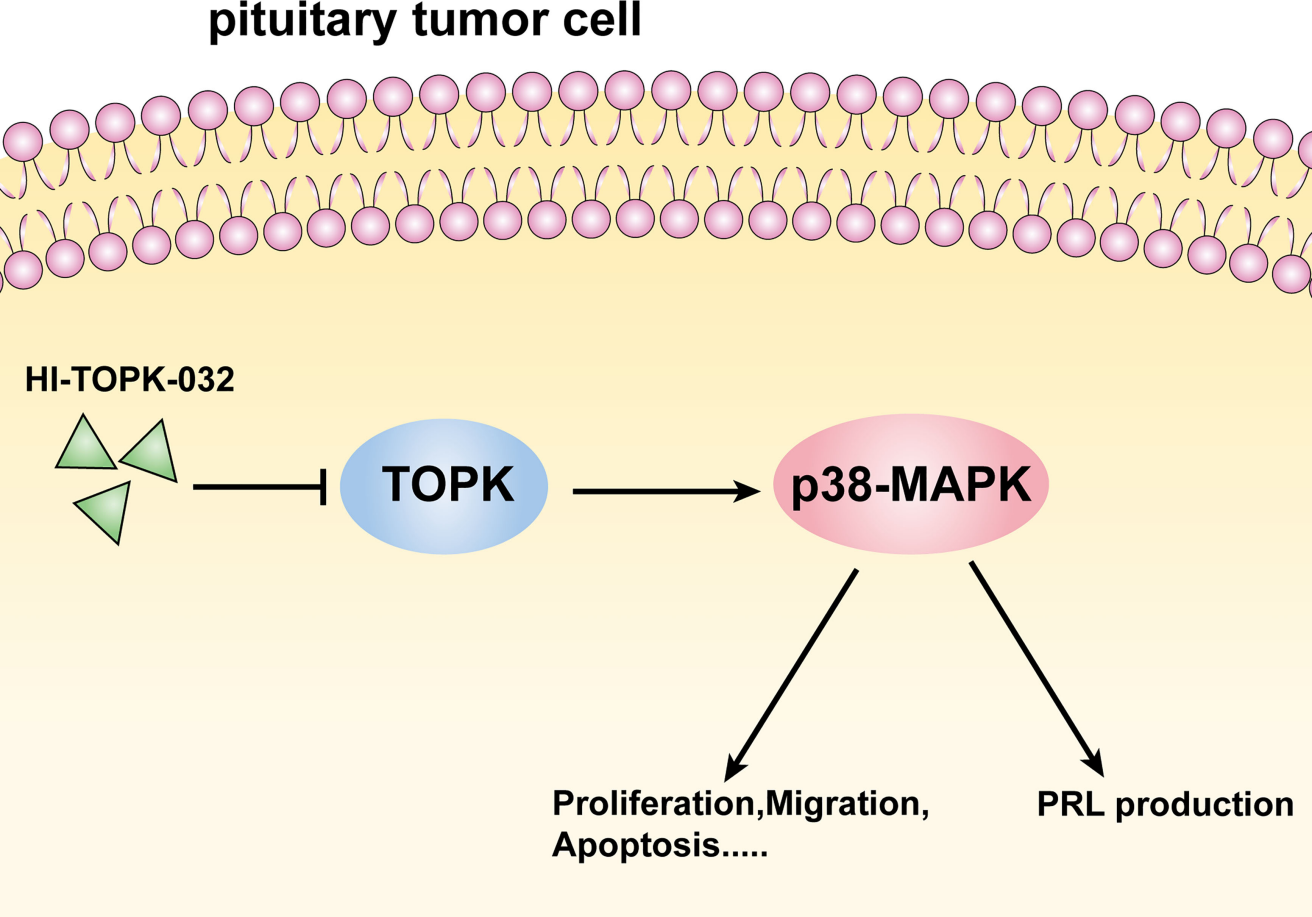
Schematic diagram of regulation mechanism.

To explore the anti-prolactinoma effect of HI-TOPK-032 *in vivo*, we established a prolactinoma model by injecting estradiol benzoate in rats. In the treatment group, rats were given HI-TOPK-032 (10 mg/kg) 3 times a week for total 28 days. The body weight changes of rats in various groups within 28 days were shown in **Figure 5A**, and the results demonstrated that HI-TOPK-032 did not cause a sharp decrease of body weight, and the body weight change in HI-TOPK-032 treatment group was similar to that in control group and model group. Compared with the control group, the prolactinoma growth was significantly increased in the model group (n = 6, *P* < 0.05); Compared with the model group, prolactinoma growth was significantly decreased in HI-TOPK-032 treatment group (n = 6, *P* < 0.05) (**Figures 5B, C**). The serum PRL level of rats in various groups was detected using PRL ELISA Kit. The results (**Figure 5D**) showed that compared with the control group, the serum PRL level was evidently increased in the model group, but such increase was significantly reversed by HI-TOPK-032 (n = 6, *P* < 0.05). As shown by the results of Western Blot assay (**Figures 5E–H**), the protein expression levels of PRL, total TOPK, p-TOPK, total p38 MAPK, and p-p38 MAPK were all higher in the model group than in the control group, while HI-TOPK-032 significantly decreased PRL protein expression level and the phosphorylation levels of TOPK and its downstream target p38 MAPK. It suggested that the protein expression of TOPK and p38 MAPK was up-regulated in prolactinoma model group, while HI-TOPK-032 declined the phosphorylation levels of TOPK and p38 MAPK, indicating that TOPK may promote the occurrence and development of prolactinoma by mediating P38 MAPK pathway. To give a brief and concise overview of the results of this paper, we draw a graphical abstract to illustrate the regulatory mechanism, as shown in **Figure 6**.

## Discussion

In recent years, the fast development of gene chip and RNA sequencing technology enables rapidly measuring the expression levels of tens of thousands of genes in various tissues and provides good tools and platforms for tumor research (41, 42). Benefiting from the opening and sharing of gene chip data and transcriptome sequencing data in various platforms, we can easily obtained the gene expression data and clinical information of tumor patients from some public databases (e.g., GEO Database) (43). PPI network was constructed by screening the DEGs between tumor tissues and normal tissues, and the DEGs-involved biological processes and signaling pathways were explored, which could provide a reliable clue for identifying the disease-related hub genes (44, 45). Therefore, identifying DEGs in prolactinoma by bioinformatic analysis provides a new hope for the development of novel treatment targets and the research of brand-new therapeutic drugs.

We searched the expression profile datasets of normal pituitary tissues and prolactinoma tissues from GEO Database and identified DEGs. Hub genes with the highest connection among these DEGs may play important roles in the development of prolactinoma. The results of differential expression analysis showed that the TOPK expression was very low in normal pituitary tissues, but significantly increased in prolactinoma tissues. Our previous research found that p38 MAPK is a candidate molecule for the pathogenesis of prolactinoma, and inhibition of p38 MAPK showed anti-prolactinoma effect (26). TOPK has also been reported as a member of MAPKK family that can selectively interact with and phosphorylate p38 MAPK (13, 46). TOPK may promote the occurrence and development of prolactinoma by mediating p38 MAPK pathway. Eventually, we used a new TOPK inhibitor HI-TOPK-032 to explore its function on the proliferation, migration and apoptosis of pituitary tumor cells and its regulation on PRL secretion. By inhibiting the activity of TOPK *in vitro*, it was found that the inhibition of TOPK could reduce proliferation and migration of pituitary tumor cells, induce apoptosis and cell cycle arrest, and decrease PRL production at the gene, protein and secretion levels. In the *in vivo* experiment, HI-TOPK-032 (10 mg/kg) inhibited the tumor growth and PRL expression & secretion in prolactinoma model rats. In addition, we also found that HI-TOPK-032 could inhibit the phosphorylation of p38 MAPK.

TOPK, as a serine-threonine kinase, belongs to the MAPKK family and is closely associated with a variety of biological activities (47). TOPK has been reported to play a role in the cell cycle regulation and mitosis process and become active during mitosis to phosphorylate Thr9 residues of CCNB1/CDK1 (48). During mitosis, PBK/TOPK makes a kinase-substrate complex with CCNB1/CDK1 and PRC1 on microtubules during mitosis, which enhances the CCNB1/CDK1-dependent phosphorylation of PRC1, thereby strongly promoting cytokinesis (49). TOPK, as a novel oncogene, plays a crucial role in the development and progression of cancer, and targeting TOPK may help provide a new method for the treatment of cancer. Therefore, it is an attractive potential target for the development of chemotherapeutic inhibitors. Through bioinformatic analysis of public datasets, we identified TOPK as a potentially important mediator of prolactinoma. Then we further confirmed that inhibition of PBK/TOPK has anti-prolactinoma effects *in vitro* and *in vivo*. This provides a new idea for the treatment of prolactinoma. However, the existing TOPK inhibitors have been reported to have some potential toxicity, such as hematotoxicity (50). Toxicity has limited the development of drugs to some extent. Therefore, how to avoid the lack of drug solubility and decrease blood toxicity must be considered. TOPK is considered to be a MAPKK-like protein that may be involved in ERK, JNK and p38 MAPK signaling pathway in a cell type-dependent manner (25). However, TOPK has not been reported to phosphorylate Tyr or Thr residues in MAPK signaling pathway to date. Therefore, further research is needed to explore the mechanism of TOPK regulating MAPKK downstream proteins.

As shown in this study, TOPK has been identified as a novel target of prolactinoma and participates in p38 MAPK signaling pathway. Although some characteristics of TOPK have not been revealed, the function of TOPK as an attractive target and valuable tumor biomarker for prolactinoma is explored. Therefore, this study provides new insights into the development of new therapeutic drugs for prolactinoma. As more compounds targeting TOPK are developed, this means that TOPK inhibitors will eventually be used in clinical settings.

## Conclusion

TOPK inhibitor HI-TOPK-032 can inhibit prolactinoma growth and PRL secretion *in vivo* and *in vitro* by mediating p38 MAPK. This study identified TOPK as a potential target for regulating the progression of prolactinoma, providing a new idea for the treatment of prolactinoma.

## Data Availability Statement

Publicly available datasets were analyzed in this study. This data can be found here: https://www.ncbi.nlm.nih.gov/geo/query/acc.cgi?acc=GSE119063.

## Ethics Statement

The animal study was reviewed and approved by Ethics Committee of Tongren Hospital affiliated to Wuhan University.

## Author Contributions

Conceptualization: XW, YC, and JW. Data curation: KZ and XC. Formal analysis: KZ and SW. Investigation: KZ and HZ. Methodology: KZ and YZ. Resources: XW, YC, and JW. Software: KZ and XC. Validation: XW, YC, and JW. Writing–original draft: KZ. Writing–review & editing: XW, YC, and JW. All authors contributed to the article and approved the submitted version.

## Funding

This work was supported by Scientific Research Project of Hubei Health Committee (grant number ZY2019Z013), Scientific Research Project Funds for Wuhan Health and Family Planning Commission (grant number WX20M02), the Special Funds for Local Science and Technology Development Guided by the Central Government (grant number 2020ZYYD026) and the Natural Science Foundation of Hubei Province (grant number 2021CFB587).

## Conflict of Interest 

The authors declare that the research was conducted in the absence of any commercial or financial relationships that could be construed as a potential conflict of interest.

## Publisher’s Note

All claims expressed in this article are solely those of the authors and do not necessarily represent those of their affiliated organizations, or those of the publisher, the editors and the reviewers. Any product that may be evaluated in this article, or claim that may be made by its manufacturer, is not guaranteed or endorsed by the publisher.

## Supplementary Material

Supplementary Figure 1 GO and KEGG enrichment analysis. **(A)** GO enrichment analysis of up-regulated genes. **(B)** GO enrichment analysis of down-regulated genes. The top ten GO terms were presented from the aspects of BP, CC and MF. **(C)** KEGG pathway enrichment analysis of up-regulated genes. **(D)** KEGG pathway enrichment analysis of down-regulated genes.

Supplementary Figure 2 HI-TOPK-032 up-regulated Bax expression but down-regulated Bcl-2 expression in pituitary cells. **(A)** Expression of Bax in MMQ cells. **(B)** Expression of Bax in GH3 cells. **(C)** Expression of Bcl-2 in MMQ cells. **(D)** Expression of Bcl-2 in GH3 cells.
