# Supplementary material for: PBK/TOPK Inhibitor Suppresses the Progression of Prolactinomas
Source: Front Endocrinol (Lausanne). 2022 Jan 21;12:706909. doi: 10.3389/fendo.2021.706909 (PMC8815076; doi:10.3389/fendo.2021.706909)
Supplement: Supplementary Figure 1 — GO and KEGG enrichment analysis. (A) GO enrichment analysis of up-regulated genes. (B) GO enrichment analysis of down-regulated genes. The top ten GO terms were presented from the aspects of BP, CC and MF. (C) KEGG pathway enrichment analysis of up-regulated genes. (D) KEGG pathway enrichment analysis of down-regulated genes. [file DataSheet_1.docx]

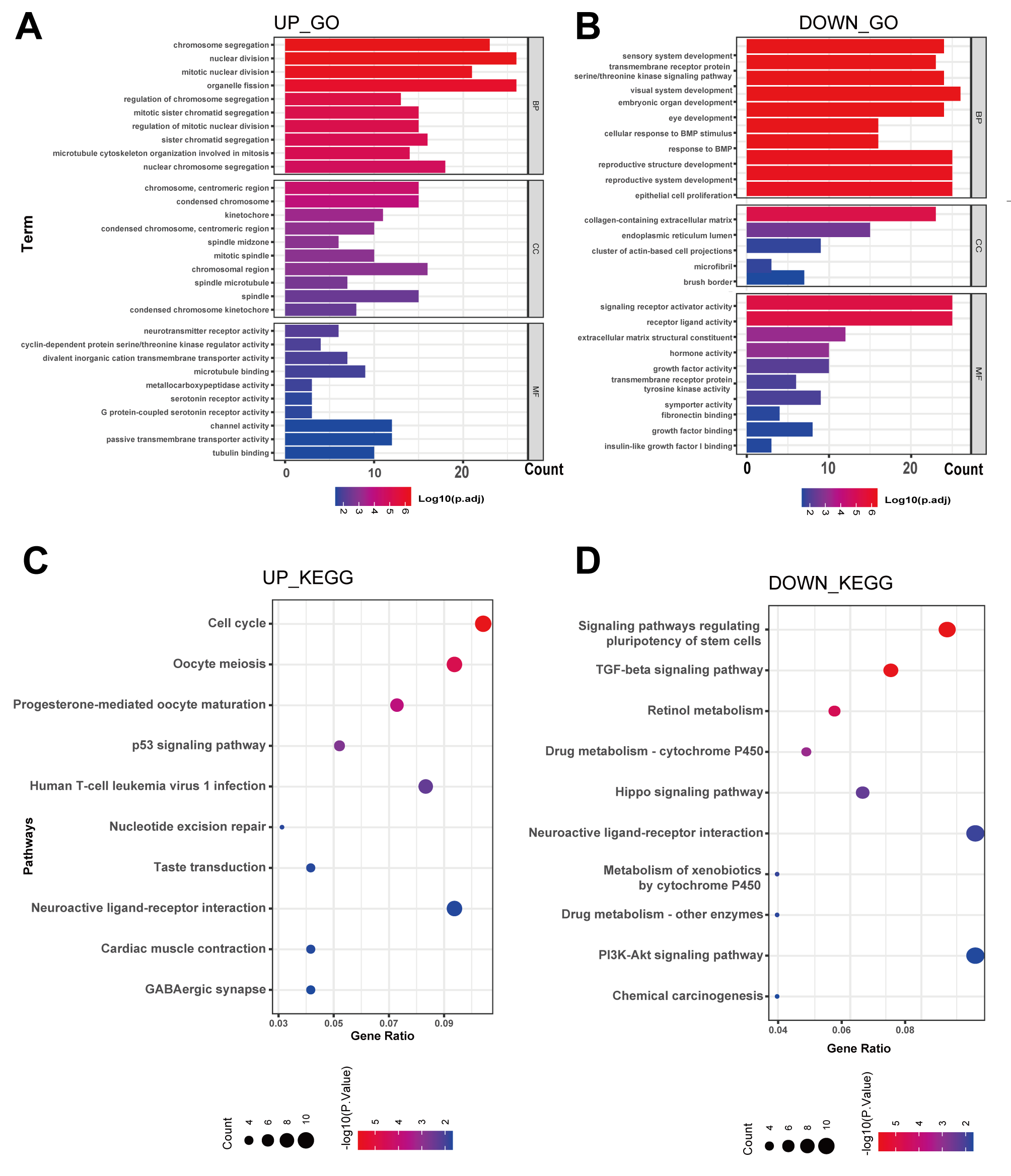


Supplementary Figure 1 GO and KEGG enrichment analysis.

(A) GO enrichment analysis of up-regulated genes. (B) GO enrichment analysis of down-regulated genes. The top ten GO terms were presented from the aspects of BP, CC and MF. (C) KEGG pathway enrichment analysis of up-regulated genes. (D) KEGG pathway enrichment analysis of down-regulated genes.


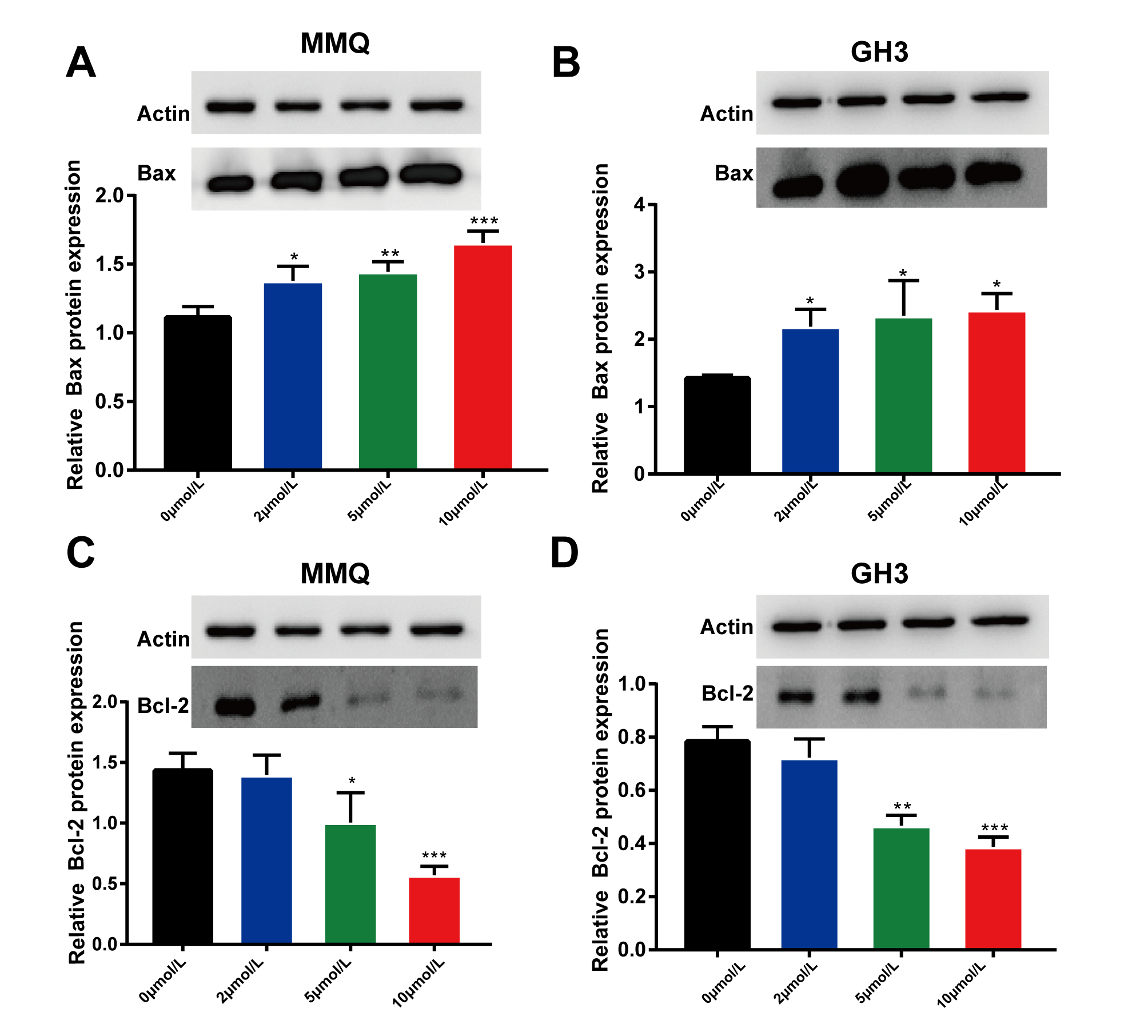


Supplementary Figure 2 HI-TOPK-032 up-regulated Bax expression but down-regulated Bcl-2 expression in pituitary cells.

(A) Expression of Bax in MMQ cells. (B) Expression of Bax in GH3 cells. (C) Expression of Bcl-2 in MMQ cells. (D) Expression of Bcl-2 in GH3 cells.
